# Cosmetic Talc–Related Pulmonary Granulomatosis

**DOI:** 10.1177/2324709617728527

**Published:** 2017-09-08

**Authors:** Sonia Jasuja, Brooks T. Kuhn, Michael Schivo, Jason Y. Adams

**Affiliations:** 1 University California Davis Medical Center, Sacramento, CA, USA; 2 University of California Davis, CA, USA

**Keywords:** cosmetic talc, granulomatosis, pulmonary nodule, foreign body, inhalational pulmonary granulomatosis, pulmonary talcosis

## Abstract

Inhalation of cosmetic talc can lead to pulmonary foreign-body granulomatosis, though fewer than 10 cases of inhaled cosmetic talc–related pulmonary granulomatosis have been reported in adults. We report the case of a 64-year-old man with diffuse, bilateral pulmonary nodules and ground glass opacities associated with chronic inhalation of cosmetic talc. Transbronchial biopsy showed peribronchiolar foreign-body granulomas. After cessation of talc exposure, the patient demonstrated clinical and radiographic improvement without the use of corticosteroids. This case demonstrates that a conservative approach with cessation of exposure alone, without the use of corticosteroids, can be an effective therapy in cosmetic talc–related pulmonary granulomatosis.

## Introduction

Talc is composed of crystallized magnesium silicate and is found in common household items such as baby powder, cosmetics, and pharmaceutical tablets.^1^ Talc is a known cause of pulmonary foreign-body granulomatosis in intravenous drug users, mainly from talc binders present in dissolved prescription drugs. Less commonly, inhalation of talc can lead to pulmonary foreign-body granulomatosis, though fewer than 10 cases have been reported in adults. Inhaled pulmonary talcosis is often treated with oral corticosteroids, though supportive data for this practice is limited.

## Case Report

**Figure 1.**
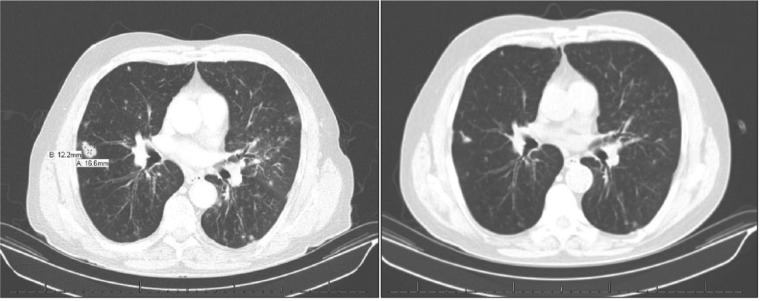
Left: CT chest on presentation. Note the diffuse poorly defined nodules and ground glass opacities. Right: CT chest 9 months after cessation of cosmetic talc use, demonstrating resolution of ground glass opacities and smaller, less dense nodules.

**Figure 2.**
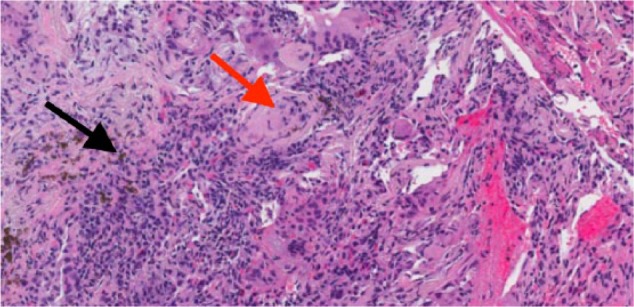
Transbronchial biopsy with hematoxylin-eosin staining showing foreign-body giant cell reaction containing round to oval polarizable material (black arrow) and foreign-body granuloma (red arrow).

We report the case of a 64-year-old Caucasian man with a 50 pack-year history of tobacco abuse and obstructive sleep apnea on nocturnal continuous positive airway pressure who presented to pulmonology clinic with indolent, worsening shortness of breath and productive cough over the preceding year. The patient was a retired auto-mechanic with previous reported asbestos exposure from car brakes. He had no other significant exposure history. Physical exam included fine rales at both lung bases. Computed tomography (CT) of the chest showed diffuse, bilateral pulmonary nodules, hilar and mediastinal lymphadenopathy, and scattered ground glass opacities, which were new in comparison to his previous chest CT 2 years prior to this encounter (Figure 1). Bronchoalveolar lavage (BAL) showed a predominance of neutrophils, as shown in Table 1. Transbronchial biopsy showed a foreign-body giant cell reaction with intracellular round to oval polarizable material (Figure 2). Our facilities did not have the capability to perform birefringence testing on a routine basis, and therefore it was not done.

**Table 1.** Bronchoalveolar Lavage.

White blood cell count (/mm^3^)	213
Neutrophils (%)	90%
Lymphocytes (%)	2%
Monocytes (%)	3%
Eosinophils (%)	3%
Histiocytes (%)	2%
Red blood cell count (/mm^3^)	11 250
Cultures (bacterial, AFB, fungal)	Samples lost

Further history revealed that the patient was applying copious amounts of baby powder to his bed-bound wife twice daily for the last year, which corresponded to the period of symptom onset and worsening. A diagnosis of inhaled cosmetic talc–related pulmonary granulomatosis (ICTRPG) was made. Over the 9 months following cessation of talc exposure, the patient demonstrated progressive symptomatic improvement without the use of corticosteroids. Repeat CT of the chest showed a decrease in the size and number of multiple bilateral pulmonary nodules, and resolution of ground glass opacities.

## Discussion

Inhaled talc pneumoconiosis was first described by Thorel in 1896.^2^ Most cases of foreign-body granulomatosis from talc are due to either occupational exposure to talc or intravenous injection of talc-containing oral tablets.^1^ There are 4 forms of talc-induced lung injury, 3 of which involve inhalation.^1^ The first form, known as talc asbestosis, occurs with inhalation of talc with asbestiform fibers, mimicking pulmonary asbestosis.^3^ During production of commercial talc, thin, elongated talc crystal cleavage fragments are created that meet the World Health Organization definition of fibers.^3^ These fibers deposit in the small airways and alveoli leading to a fibrotic reaction over time. The second form, talcosilicosis, occurs after talc with a high silica content (a by-product of mining talc) is repeatedly inhaled over time. Clinically, talcosilicosis is indistinguishable from silicosis, producing nodules and, occasionally, fibrosis involving the mid- to upper-lung zones.^4^ The third and rarest form, talcosis, occurs with inhalation of pure talc (described in more detail below). The fourth form, seen after intravenous use of crushed opiate pills, leads to perivascular pulmonary granulomas.^5^ These granulomas are thought to result from a delayed hypersensitivity reaction that manifests as epithelioid cells and foreign-body giant cells.^6^

Inhalation of cosmetic talc presents with a wide variety of nonspecific clinical symptoms including dyspnea on exertion, cough, chest pain, weight loss, anorexia, low-grade fevers, and night sweats.^1,7-9^ Symptoms typically develop shortly after exposure, but can manifest up to 30 years later posing a significant diagnostic challenge.^1^ Pulmonary hypertension, emphysema, and chronic respiratory failure may occur after long-term exposure.^10^ In some cases, death attributed to talc exposure may occur.^7^

Inhalation talcosis typically demonstrates solid nodules and ground glass opacities distributed throughout all lung zones on chest CT.^7,10^ In contrast, talcosilicosis and talc asbestosis appear indistinguishable from silicosis and asbestosis, respectively. Imaging of patients with talc granulomatosis from intravenous injection can show dense nodules, masses, and lower lobe predominant emphysema.^7,10^ All forms of talcosis can lead to pulmonary fibrosis.

ICTRPG pathology demonstrates nonnecrotizing granulomas with multinucleated foreign-body giant cells containing birefringent foreign bodies under polarized light.^7,11^ ICTRPG-associated foreign-body granulomas are typically found surrounding small airways and alveolar septae, with larger nodules resulting from the coalescence of granulomas in chronic disease.^7^ In contrast, foreign-body granulomatosis from intravenous injection of talc (eg, from dissolved opioid pharmaceutical tablets) results in granuloma formation in a perivascular pattern that spreads into the interstitium. Crystals are larger in intravenous exposure compared with inhalational exposure.^7^ Ultimately, interstitial fibrosis can develop in both forms of talc exposure.^7^ In all cases, it is important to exclude other causes of nonnecrotizing granulomas with or without foreign bodies, including chronic aspiration and sarcoidosis. The clinical history of inhalational talc exposure, absence of alternative diagnoses, and a normal swallowing mechanism are usually sufficient to make the diagnosis of ICTRPG.

Numerous cases of ICTRPG have demonstrated persistent granulomatosis and pulmonary fibrosis despite cessation of exposure to cosmetic talc.^9,12,13^ Cruthirds et al described a 2-year-old patient with a massive talc aspiration leading to pulmonary fibrosis. Despite functional improvement, the patient subsequently developed pulmonary hypertension 16 years after the exposure event.^12^ van Huisstede et al described a patient with perceived improvement, although her imaging showed stable, unchanged nodular lesions in bilateral lung fields despite cessation of cosmetic talc use.^13^ In contrast, our case demonstrates both symptomatic and radiographic improvement with cessation of exposure, an outcome that has not been documented in previous cases of ICTRPG.

Corticosteroids are used to treat ICTRPG based on case reports and expert opinion.^8,14^ Iqbal et al described a case of ICTRPG in which the initial use of prednisolone resulted in symptomatic improvement; however, cessation of the corticosteroid led to recurrence of symptoms indicating that talc exposure was ongoing or that talc-related inflammation was not fully suppressed.^6^ Conversely, Ong and Takano described a case of ICTRPG in which the use of prednisolone with a slow taper resulted in improvement in symptoms, lung function, and radiographic findings.^14^

An apt comparison for ICTRPG is with extrinsic allergic alveolitis, also known as hypersensitivity pneumonitis (HP). While talc is not traditionally considered one of the antigens leading to HP, ICTRPG resembles HP in many respects. Both are immune reactions to inhaled antigens leading to bronchiolocenteric nonnecrotizing granulomas. In addition, both have acute and chronic forms, with radiographic findings of ground glass opacities and centrilobular nodules.^15^ While HP and ICTRPG are different immunologically, their clinical presentations are almost identical, as demonstrated in Table 2. The pathogenesis of HP is thought of as a 2-hit model where a genetically predisposed patient is exposed to an antigen, leading to pneumonitis and possibly pulmonary fibrosis.^15^ In acute HP, the disease process is mediated by an immune complex response. In subacute and chronic HP, the disease process is mediated by T-lymphocytes via a Th1 response cascade.^15,16^ Conversely, the immunologic pathogenesis of ICTRPG is not well understood, but is thought to be a hypersensitivity reaction leading to granuloma formation via foreign-body giant cells and macrophages.^6,9,13,14^ Corticosteroids may accelerate improvement in lung function for acute HP but long-term survival benefit has not been proven.^17^ The most effective intervention in HP remains cessation of exposure to the inciting antigen.^15,17^

**Table 2.** A Comparison Between Talc Pneumonitis and Acute Hypersensitivity Pneumonitis.

	Talc Pneumonitis	Acute Hypersensitivity Pneumonitis
Symptoms	Dyspnea, nonproductive cough	Dyspnea, nonproductive cough
Chest radiography	Peribronchial reticulonodular infiltration, sparing of apices, and costophrenic angles^13^	Peribronchial reticulonodular infiltration (patchy or diffuse)
High-resolution computed tomography scan	Scattered ground glass opacities, nodules	Scattered ground glass opacities, nodules
Pulmonary function test	Restrictive pattern	Restrictive pattern
Bronchoalveolar lavage	Neutrophilic predominance	Lymphocytic predominance
Pathology	Nonnecrotizing granulomas with multinucleated foreign-body giant cells containing birefringent foreign bodies under polarized light surrounding small airways and alveolar septae	Loosely formed nonnecrotizing granulomas

Our case suggests overlap between these 2 diseases in terms of symptoms and radiographic findings, with the notable exception of the neutrophilic predominance in the BAL fluid (Table 1). Bronchoscopy in HP typically shows a T-lymphocyte predominance on BAL, which is highly sensitive but poorly specific for the diagnosis of acute HP.^15,18^ While a high antigen load or coexistent infection may cause an airway neutrophilia on BAL in HP, infection was not found in our patient.^19^ Beck et al examined the effects of inhaled talc and granite in a hamster model. Both talc and granite led to high alveolar neutrophil and decreased macrophage levels, although the effect was more transient with talc.^20^

The limitations of our case include inability to perform birefringence and lack of culture data from BAL because the samples were lost. Clinically, the patient did not appear infected and he improved without antimicrobial treatment. Furthermore, after 9 months of follow-up, the patient failed to return to clinic limiting our ability to assess longer term outcomes. We also do not know what brand of cosmetic talc he was using, and therefore, we do not know if the inhaled talc contained asbestiform particles. However, asbestiform contamination is unlikely given imaging that was not consistent with talcoasbestosis.

In our patient, we chose to remove the inciting agent without pharmacologic intervention given the risks of corticosteroid use in the context of his comorbidities. Our patient did not demonstrate radiographic evidence of fibrosis— a described sequelae of talc inhalation—possibly contributing to the reversibility of his disease. To our knowledge, this is the first published case of ICTRPG successfully treated solely with antigen removal, and it is likely that other cases of mild to moderate disease may be managed in a similar way.

## Conclusion

Our case demonstrates that cessation of cosmetic talc exposure, without the use of corticosteroids, can be an effective therapy in ICTRPG without fibrosis. While ICTRPG shares many characteristics with HP, this case shows that ICTRPG may present with a predominance of alveolar neutrophils on BAL, possibly a direct result of either recent or frequent talc exposure.
